# Poster Session II - A279 AN ANALYSIS OF LONG TERM OUTCOMES IN CROHN’S PATIENTS POST–BALLOON ASSISTED ENDOSCOPIC STRICTURE DILATION: FACTORS ASSOCIATED WITH SURGERY AND A NEW PREDICTIVE MODEL

**DOI:** 10.1093/jcag/gwaf042.279

**Published:** 2026-02-13

**Authors:** B Halloran, K Oguro, J C Bowron, J Reeve, Q Tan, D E Parsons, F Peerani, M Gozdzik, K Wong, K Kroeker, F Hoentjen, S Wasilenko, S Zepeda Gomez

**Affiliations:** University of Alberta, Edmonton, AB, Canada; University of Alberta, Edmonton, AB, Canada; University of Alberta, Edmonton, AB, Canada; University of Alberta, Edmonton, AB, Canada; University of Alberta, Edmonton, AB, Canada; University of Alberta, Edmonton, AB, Canada; University of Alberta, Edmonton, AB, Canada; University of Alberta, Edmonton, AB, Canada; University of Alberta, Edmonton, AB, Canada; University of Alberta, Edmonton, AB, Canada; University of Alberta, Edmonton, AB, Canada; University of Alberta, Edmonton, AB, Canada; University of Alberta, Edmonton, AB, Canada

## Abstract

**Background:**

Crohn’s disease (CD) is characterized by relapsing transmural inflammation of the gastrointestinal (GI) tract affecting the small bowel in > 80% of patients. One of the hallmarks of CD is intestinal stricture that may necessitate surgical resection. Although the efficacy of endoscopic balloon dilation (EBD) using balloon-assisted endoscopy (BAE) has been reported in several studies, some patients still require surgical intervention and the factors leading to surgery remain not well understood.

**Aims:**

Our objective was to identify the factors predicting the need for surgery in patients with CD that have previously undergone balloon dilation of small bowel strictures.

**Methods:**

A retrospective review was conducted at a quaternary centre (University of Alberta Hospital). Patients over 18 years of age who have undergone one or more BAE with stricture dilation for CD between January 2012 and January 2024 were included. We identified the patients from this cohort that underwent surgery for symptoms relapse or persistence of symptoms despite dilation. Univariate and multivariate logistic regression analysis were performed to identify factors predicting need for surgery.

**Results:**

A total of 136 patients were included in this study. Of those, 47 patients underwent surgery (34.6 %). The average number of BAE performed per patient was 3.57. Univariate logistic regression analysis showed the number of BAEs performed (odds ratio: OR = 1.24, p = 0.022), active inflammation (OR = 1.67, p = 0.001), minimum dilation diameter (OR = 0.74, p = 0.001), and non-traversable stricture (OR = 3.61, p = 0.004) were significantly associated with the need for surgery. Notably, the number of strictures dilated in a patient was not significant (p = 0.13). In multivariate logistic regression modelling the variables of significance were age, disease duration, biologics use, active lesions, minimum dilation diameter, and non-traversable stricture. This model had a bootstrap-corrected AUC of 0.73. A cutoff of approximately 14 mm as the minimum achieved dilation diameter was the best predictor of who would need surgery.

**Conclusions:**

A combination of patient’s demographics and stricture characteristics seems to be effective for predicting the need for surgery. Furthermore, a minimum dilation diameter greater than 14 mm may be an important endoscopic target for avoiding surgical intervention. A prospective study is currently being designed to validate the results from this patient cohort.

**Funding Agencies:**

None

